# Developing informative microsatellite makers for non-model species using reference mapping against a model species’ genome

**DOI:** 10.1038/srep23087

**Published:** 2016-03-15

**Authors:** Chih-Ming Hung, Ai-Yun Yu, Yu-Ting Lai, Pei-Jen L. Shaner

**Affiliations:** 1 Biodiversity Research Center, Academia Sinica, Taipei, Taiwan; 2 Department of Life Science, National Taiwan Normal University, Taipei, Taiwan

## Abstract

Microsatellites have a wide range of applications from behavioral biology, evolution, to agriculture-based breeding programs. The recent progress in the next-generation sequencing technologies and the rapidly increasing number of published genomes may greatly enhance the current applications of microsatellites by turning them from anonymous to informative markers. Here we developed an approach to anchor microsatellite markers of any target species in a genome of a related model species, through which the genomic locations of the markers, along with any functional genes potentially linked to them, can be revealed. We mapped the shotgun sequence reads of a non-model rodent species *Apodemus semotus* against the genome of a model species, *Mus musculus*, and presented 24 polymorphic microsatellite markers with detailed background information for *A. semotus* in this study. The developed markers can be used in other rodent species, especially those that are closely related to *A. semotus* or *M. musculus*. Compared to the traditional approaches based on DNA cloning, our approach is likely to yield more loci for the same cost. This study is a timely demonstration of how a research team can efficiently generate informative (neutral or function-associated) microsatellite markers for their study species and unique biological questions.

Microsatellites have been applied to a wide range of biological studies given their extensive genome distribution, high level of polymorphism, and high amplification success^1^. However, microsatellite markers mostly are anonymous DNA fragments, obstructing their usefulness in deeper applications^2^. Long considered as neutral markers, microsatellites have been used for parentage analysis, population genetics, and natural resource management^3^,^4^,^5^,^6^. More recently, studies have found that a significant portion of microsatellites are associated with functional changes, and mutations that may cause diseases^7^,^8^; furthermore, some are so closely linked to genes under selection that they deviate from neutral patterns^9^,^10^. Consequently, microsatellites can be used to tag corresponding functional genes or map quantitative trait loci (QTL)^11^,^12^. These characters make microsatellites useful markers beyond their early application in population genetics. However, the conventional protocols based on DNA cloning generate microsatellite markers without background information on genomic locations or associated gene functions, both of which are important in the study of nature selection or selective sweeps and the evolution of agriculturally or medically important traits^1^.

There have been several new approaches that use the next-generation sequencing (NGS) technologies to develope microsatellite markers making the procedures more efficient in terms of time and money and potentially broadening its application in biology^13^,^14^,^15^,^16^,^17^. However, genomic background information of most markers is still unavailable. Even though microsatellite markers associated with functional genes can be developed from expressed sequence tags (ESTs) or transcriptomes, such approaches can only generate markers mostly located in coding regions, representing a minor part of a genome^12^,^15^. By contrast, whole genomes published for a variety of species have provided unprecedented resources to develop genome-wide microsatellite markers with detailed genomic background, even for species whose genomes are not likely to be assembled in the foreseeable future.

Rodents have been the subjects of study in a wide array of biological disciplines, from evolutionary biology, to community ecology and epidemiology given their widespread distributions and close interactions with humans^18^,^19^,^20^,^21^,^22^. The house mouse (*Mus musculus*) can provide valuable reference information for developing microsatellite markers for other rodents because its whole genome sequences and gene ontology are well studied^23^. In this study, we developed microsatellite markers with annotation such as distances between microsatellite motifs and surrounding functional genes for a rodent species, *Apodemus semotus*, by mapping its raw sequence reads against the *M. musculus* genome. The two genera *Apodemus* and *Mus* have diverged *c*. 10 million years ago^24^. *Apodemus* are the most common rodents in the temperate zone of the Palearctic region^25^, and may show diverse adaptations throughout their wide distribution range via natural selection. The markers developed here can also be used in other species closely related to *A. semotus* or *M. musculus.*

## Methods

### Genomic DNA extraction and genome sequencing

One notch (~1.5 mm diameter) of ear tissue was cut using an ear punch from each of 24 *A. semotus* individuals collected at the Shei-Pa National Park in Taiwan. The tissue samples were preserved in 95% EtOH and stored in a −20 °C freezer until DNA extraction. Genomic DNA was extracted from the tissue samples using the LiCl method^26^.

### Ethics statement

We had IACUC approval for rodent trapping and tissue sampling procedures in this project through National Taiwan Normal University’s Institutional Animal Care and Use Committee (protocol no. 102004); all processes involving live animals were performed in accordance with the approved guidelines.

### Genome sequencing and Reference mapping

Illumina shotgun sequencing was applied to the genomic DNA of a female *A. semotus* sample. An Illumina library with an insertion size of 500 bp was prepared from 2.74 μg of DNA. Paired-end 90 bp sequence reads were obtained from a lane of Illumina HiSeq 2000. The library preparation and sequencing were performed by Beijing Genomic Institute (BGI, Shenzhen, China).

The whole genome sequences of *M. musculus* (GRCm38.p3, downloaded from the NCBI GenBank) was used as the reference genome. The Illumina paired-end reads of *A. semotus* were mapped against the genome of *M. musculus* using CLC Genomics Workbench 6.02 (CLC Inc, Aarhus, Denmark). The “Reference Mapper” tool of CLC was run with the default parameter settings (insertion cost  =  3, deletion cost  =  3, mismatch cost  =  2, length fraction  =  0.5) except that “similarity fraction” was changed from 0.8 to 0.85 to increase the portion of conserved regions in the mapped genome.

### Identification of microsatellite loci and primer design

Fragments of mapped sequences (hereafter “scaffolds”) were screened using MSATCOMMANDER 1.0.8^27^ to identify tetra-microsatellite motifs with a minimum of 8 tandem repeats and to design primers with a maximum product size of 450 bp. The primers were designed using Primer3^28^ implemented in MSATCOMMANDER. The primer size was set from 20 to 24 bp, the “T_M_” was set at 57 °C (with a range of 54–65 °C), and the other parameters were set as default. A M13R (5′-GGAAACAGCTATGACCAT-3′) or CAG tag (5′-CAGTCGGGCGTCATCA-3′) was added on the 5′ end of one primer from each pair to enable the application of a third primer that was fluorescently labeled with FAM, HEX, TEMRA.

### Microsatellite amplification and genotyping

Polymerase chain reactions (PCRs) were performed in 10 μl reaction mixture, containing 15–25 ng DNA, 0.05 μM of each primer, 0.5 μM of each primer with a M13R or CAG tail, 0.5 μM of a fluorescently labeled M13R-tag or CAG-tag primer, 0.2 mM of each dNTP, and 0.75U *Taq* polymerase (TOYOBO, Blend Taq -Plus-) with 1X PCR buffer. The PCR cycling profile was consisted of an initial denaturation step of 2 min at 95 °C followed by 20 cycles of 95 °C for 30 s, 60 °C (decreased 0.5 per cycle) for 30 s and 72 °C for 40 s and 20 cycles of 95 °C for 30 s, 50 °C for 30 s and 72 °C for 40 s, followed by a final extension step at 72 °C for 7 min. Amplified microsatellite products were genotyped using ABI 3730XL sequencer (Applied Biosystems), and allele sizes were scored using PeakScanner (Applied Biosystems).

### Genotype data analysis

The program CERVUS 3.0^29^ was used to estimate the number of alleles (N_A_), expected (H_E_) and observed heterozygosity (H_O_). We used GENEPOP 4.2^30^ to assess deviation from Hardy-Weinberg equilibrium (HWE). MICRO-CHECKER ver. 2.2.3^31^ was used to test for null alleles.

### Identifying protein genes linked to microsatellites

We firstly compared *A. semotus* DNA sequence fragments (i.e., query fragments) that extend from 100 kbp upstream to 100 kbp downstream of the regions flanked by microsatellite primers against *M. musculus* protein sequences (GRCm38.p3, downloaded from the NCBI GenBank) using BLASTX v 2.2.30+^32^,^33^ with a threshold E-value of 10^−6^. The region of 200 kbp was chosen because it was a reasonable range that a selective sweep might affect^9^. Secondly, we used NCBI Map Viewer^34^ (http://www.ncbi.nlm.nih.gov/mapview/) to locate genes with annotated intron and exon structures in the 200 kbp regions (from 100 kbp upstream to 100 kbp downstream) centering the microsatellites in the *M. musculus* genome. We considered the genes potentially linked to the microsatellites in *A. semotus* scaffolds only when they were identified in both the BLASTX and Map Viewer results (Supplementary Fig. S1). In other words, the Map Viewer results were used to filter the BLASTX outputs for *A. semotus* to avoid false positive results.

In the Map Viewer results, predicted or uncharacterized genes were labeled as “Gm”, “LOC” or “Rik” and microRNA as “Mir”. However, the BLASTX approach could not find these genes. For the sake of simplicity, we did not take them in to account in this study.

### Cross-species amplification test

We used 13 other rodent species (*A. agrarius, M. musculus, M. caroli*, *Micromys minutus, Niviventer coxingi*, *N. culturatus, Bandicota indica, Rattus exulans, R. losea*, *R. norvegeicus, R. tanezumi, Microtus kikuchii* and *Eothenomys melanogaster*) from eight genera to test cross-species amplification for the primers designed from *A. semotus*. The 13 species and *A. semotus* have diverged over 20 million years^35^. Five samples from each of the 13 species were used to estimate the successful amplification rates of the microsatellite loci. A sample would need to amplify a product of the expected size with a lack of smearing to be considered successful.

## Results

### Mapped genome of *A. semotus*

We started with 32.6 Gb paired-end sequence reads of *A. semotus*. By mapping the reads against the *M. musculus* genome with a size of 2.72 Gb, we obtained 271 scaffolds with a total size of 1.7 Gb (excluding mapping gaps) and an average sequencing coverage (or depth) of 5.6-fold.

### Microsatellite screening and quality evaluation

We identified 63,672 tetra-repeat microsatellite motifs in 90 scaffolds longer than one million bp. We designed primers for 1,456 microsatellite motifs. We randomly chose 2–5 microsatellite loci from the scaffolds corresponding to each chromosome (except for the 7th and sexual chromosomes) of *M. musculus*. This led to a total of 59 loci (see Supplementary Table S1 and Fig. S1 for detailed genomic locations) for us to test their amplification rates and polymorphism in *A. semotus*. We used 24 *A. semotus* samples for the test. We successfully amplified 44 loci, for which more than 80% of the 24 samples could be amplified (Supplementary Table S1). Among the 44 loci, 24 displayed polymorphism and could be clearly scored with no ambiguous peaks in size profiles (mean N_A_ = 7.4, mean H_E_ = 0.689, mean H_O_ = 0.599, 18 loci were in HWE; Table 1).

### Protein genes potentially linked to microsatellites

**Figure 1:**
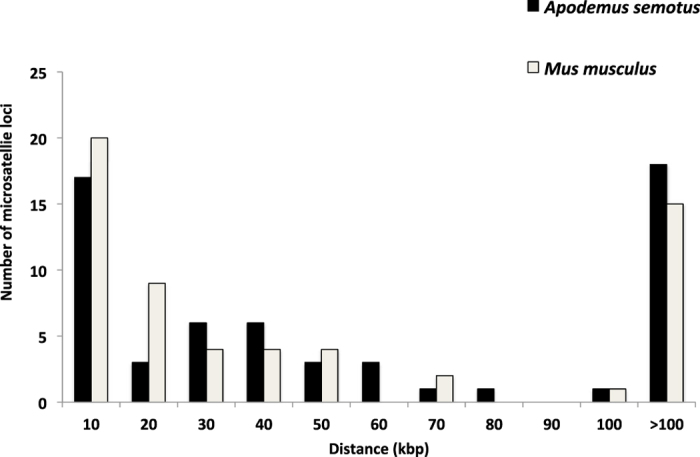
Distribution of the distance between a microsatellite locus and its nearest exon in the *Mus musculus* genome (identified using Map Viewer) or between a microsatellite locus and its nearest coding region in *Apodemus semotus* scaffolds (identified using BLASTX).

**Figure 2:**
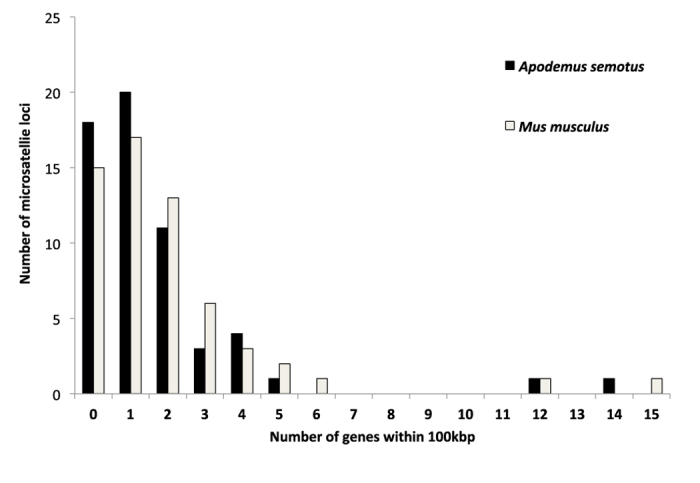
Distribution of the number of genes located within 100 kbp (upstream and downstream) from a microstatellite locus. Genes in the *Mus musculus* genome are identified using Map Viewer and that in *Apodemus semotus* scaffolds are identified using BLASTX.

Of the 59 microsatellite loci, nearly 70% (41 loci) were less than 100 kbp from the closest exon or coding region along the *A. semotus* scaffolds (Fig. 1). In the 200-kbp region centering each of the 59 loci along the *A. semotus* scaffolds, 34% (20 loci) included one protein coding gene, 19% (11 loci) included two genes and 17% (10 loci) included three or more genes (Fig. 2). The patterns found in the *A. semotus* scaffolds (based on BLASTX results) were similar to those in the *M. musculus* genome (based on Map Viewer results; Figs 1 and 2; Supplementary Fig. S1). Given that a selective sweep might affect neutral genes up to 100 kbp away from a selected one^9^, genes in the 200 kbp region can be considered potentially linked to the microsatellites (Supplementary Fig. S1).

### Cross-species amplification testing

**Figure 3:**
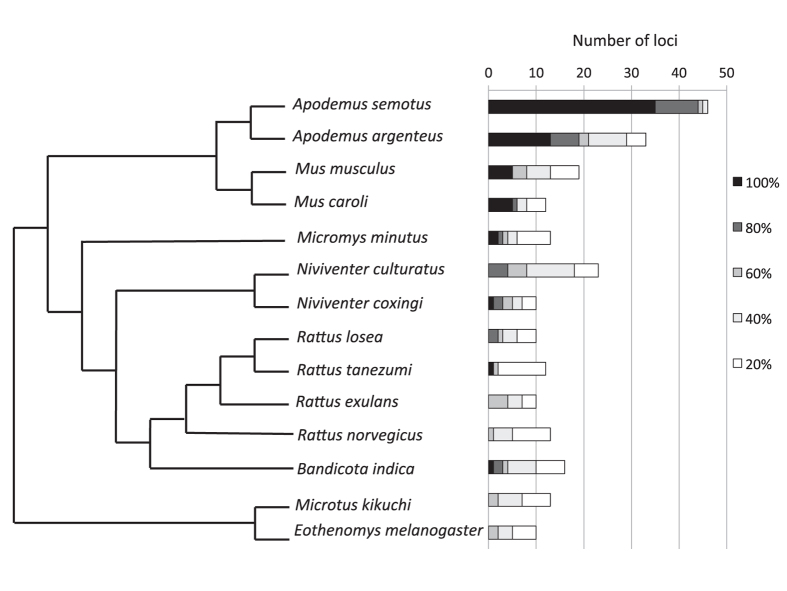
Numbers of amplified loci. A total of 59 loci generated from *Apodemus semotus* scaffolds are tested on 14 rodent species. Darker color indicates higher amplification success rates of loci, which are estimated based on 24 samples for *A. semotus* and five samples for each of the other 14 species. The cladogram (modified from Fabre et al. 2012) on the left indicates the phylogenetic relationship among these species.

At a success rate of 80% or better, we amplified 0 to 19 out of the 59 microsatellite loci across the 13 rodent species; at a success rate of 40% or better, we amplified 2 to 29 loci across the rodent species (Fig. 3 and Supplementary Table S1). A congener to *A. semotus, A. agrarius* had the highest amplification rate regardless the success rate threshold. Species that are closely related to *A. semotus* or *M. musculus* had higher success rates than others (Fig. 3).

## Discussion

We successfully devised a reference genome mapping approach to develop microsatellite makers detailed with genomic locations and potentially linked genes, for a non-model rodent species *A. semotus*. For any species, to which the genome of a closely related species is available, this approach can efficiently generate informative microsatellite markers that have a wide range of applications from behavioral biology, adaptive evolution, to agriculture-based breeding programs.

### An effective and economical approach to microsatellite development based on NGS

Our approach based on reference genome mapping is more efficient in terms of money and time than traditional approaches based on cloning of genetic libraries and Sanger sequencing. In general, traditional approaches require one to four weeks of bench work followed by Sanger sequencing^36^, whereas our approach requires only one to two days of bench work for DNA extraction followed by shotgun sequencing. The total cost for bench work and sequencing (i.e., including the cost for processing through primer design but excluding PCR test) are similar between traditional (1,100 ~ 4,400 USD)^36^ and our approaches (3,000 USD). However, the higher yield of loci of our approach (1,456 loci with at least eight tetra-nucleotide repeats) compared with the traditional ones (100 loci assuming a 50% of positive rate based on 200 screened clones)^36^,^37^ makes the former at least 5 times more cost-effective on a per-locus basis (Supplementary Table S2). Moreover, our approach can improve the quality of microsatellite makers with detailed genomic background information, which is more difficult to achieve using the traditional approaches.

Regarding the applications of different NGS technologies in microsatellite development, Illumina approaches are more cost-effective than 454 approaches^14^,^38^,^39^. Nevertheless, Illumina raw reads are usually too short to cover the entire microsatellites or to have sufficient flanking sequences for primer design^38^. Even though the combination of two paired-end reads can partially solve the problem, it provides little information on the exact number of repeats in microsatellite loci^39^. In addition, the total length of microsatellite loci developed from either Illumina or 454 raw reads is generally short^37^,^39^.

Although *de novo* assembling raw reads into longer contigs can increase the number and length of isolated microsatellite makers^14^,^17^, the required amount of raw reads and computational power are not trivial. By contrast, mapping raw reads against the reference genome of a closely related species is more cost-efficient^40^,^41^. Given the rapid accumulation of whole genome data, soon most species will have reference genomes from species close enough for genome mapping. Here we devised an Illumina-based reference mapping approach to isolate microsatellite markers, which requires minimum laboratory and computing resources and thus is affordable for most research groups. Although the mapping-based genome of *A. semotus* (1.7 Gb scaffolds with an average sequencing coverage of 5.6X) is neither complete nor high-quality, and reference-guided genomes with similar levels of coverage have been found to miss a portion of microsatellites^41^, this genome still provides thousands of primers, which is sufficient for most of the biological questions. In fact, the development of informative microsatellite makers can become a routine by-product of a genome re-sequencing (mapping) project, adding values to the core genome product.

Furthermore, aligning the microsatellite markers developed for non-model species against the reference genome of a model species can help the former to adopt annotated gene information from the latter. In general, the distributions of protein coding genes surrounding the microsatellites in *A. semotus* scaffolds (based on the BLASTX results) reflect well those in the *M. musculus* genome (based on the Map Viewer results) despite some discrepancies (Supplementary Fig. S1). Several reasons could explain the observed discrepancies: (1) the gene sequences in the two species had differentiated too much to be detected by the BLASTX approach, (2) CLC mapping gaps or errors had caused the failure to detect these genes or exons, (3) the *M. musculus* protein database was incomplete and/or (4) the Map Viewer results overestimated the presence of coding regions. Some sequence regions in the Map Viewer results might be erroneously identified as coding regions (e.g., light green vertical lines in Supplementary Fig. S1) because the Map Viewer results were merged from multiple assembles and coding regions, some of which might not have been validated. Nevertheless, the discrepancies occurred infrequently and did not substantially impact the applications of these markers.

The markers developed in this study can also be used in other rodent species closely related to either *A. semotus* or *M. musculus*. Although these microsatellites do not have universal amplification success across all 14 test species, strong cross-amplification between the two genera *Apodemus* and *Mus* that have diverged *c*. 10 million years ago^24^ is encouraging. By carefully choosing reference species (as distinct as possible but still close enough for mapping), we believe that this approach can generate makers with high cross-amplification success given the more conserved regions used for primer design.

### Applications of informative microsatellite markers

The anonymity of microsatellites is sometimes overemphasized in population genetic analysis. Although anonymous microsatellites reflect randomness in sampling, informative microsatellite markers generated by our approach can have wider and deeper applications. For example, positive selection or selective sweeps have been found in some microsatellites^9^,^10^,^42^, but the lack of information about the potential protein coding genes associated with these microsatellites prevents further understanding of the selection scenarios. Our approach is desirable in that it allows identification of functional genes linked to microsatellites. Even though informative microsatellites have been developed from expressed sequence tags (ESTs) or transcriptomes, such approaches can only generate markers that are mainly located in coding regions and the EST microsatellites are less polymorphic and less representative of genome-wide patterns^12^,^15^. By contrast, our approach is more flexible in that genome-widely distributed microsatellite loci with different levels of linkage to protein coding genes can be developed, which provide rich materials for testing positive selection and selective sweeps^9^.

Given there is a wealth of quantitative trait loci (QTLs) with genome coordinates available in public databases for model species (e.g., Mouse Genome Informatics database; http://www.informatics.jax.org), our approach can also be applied to develop microsatellite markers near the QTLs of interest for non-model species (e.g., *Apodemus* and other *Mus* species). Such markers provide useful tools to investigate the genetic basis of quantitative phenotypic traits. Furthermore, the whole genome or QTL database of the study species *per se* is not required using our approach, making it widely applicable across species.

On the other hand, for genetic analyses that require neutral markers, such as effective population size estimation, our approach is useful in *a priori* filtering out makers that are closely linked to functional genes (e.g., microsatellites located in exonic or intronic regions of protein coding genes) and thus can potentially deviate from neutral patterns, which may improve the quality of the genetic inferences. Our approach also allows ones to choose independent markers that are widely distributed across the whole genome to avoid marker-biases in population genetics analysis.

The application of microsatellites in population genetics has been criticized due to the concern of size homoplasy^43^, null alleles^44^, or artificial population structure^45^,^46^ although some argue that they could perform better than single-nucleotide polymorphism (SNP) data in recovering recent population structure^47^. Aside from the controversial role of microsatellites in the studies of population genetics, the applications of microsatellites remain very useful in behavioral biology. For example, researchers can identify sex-linked loci using our approach to maximize the confident level of paternal assignment in mammals given that Y-chromosome loci have higher resolution than autosomal ones in identifying fathers of male mammalian individuals. An increase in precision of parentage assignment through informative microsatellite markers can enhance our ability to understand the selection processes underlying animal behaviors, such as extra-pair mating or conspecific brood parasitism.

## Conclusion

In the NCBI Genbank database (February 2016), there are already 116 mammal genomes, 66 bird genomes, 66 fish genomes, 194 insect genomes, 159 land plant genomes, just to name a few. For mammals, 17 rodent genomes are available; for birds, almost every order has at least one genome available. There are more ambitious, ongoing genome projects, such as Genome 10K^48^, i5K^49^ and B10K^50^ projects, which will generate thousands of genomes for several taxonomic groups in the near future. Our study is a timely demonstration of how to utilize the growing genome database to reenergize existing genetic tools. Even though most published genomes have not been studied as explicitly as the house mouse genome, they are still enough to develop annotated microsatellite makers. The mapping approach can also be applied to extinct species^51^ to isolate microsatellite markers for them. As the DNA extracted from fossils or old specimens is highly fragmented, microsatellites characterized by small lengths are ideal markers to study extinct species. In conclusion, this study highlights our untapped power to generate “custom” genetic markers in the genomics era.

## Additional Information

**Accession codes:** Illumina short reads deposited in NCBI Genbank under accession number SRP071097. 

**How to cite this article**: Hung, C.-M. *et al*. Developing informative microsatellite makers for non-model species using reference mapping against a model species' genome. *Sci. Rep.* **6**, 23087; doi: 10.1038/srep23087 (2016).

## Supplementary Material

Supplementary Information

Supplementary Information

## Figures and Tables

**Table 1:** Characteristics of 24 microsatellite loci genotyped in *Apodemus semotus*.

Locus	Ch	Primer sequence	Motif	N	Size	N_A_	H_E_	H_O_	P_HWE_	Protein coding gene
1A720	1	F-GATAGACATCTCAGTGCCAAAC	(AAAG)^14^	22	335–345	4	0.354	0.318	0.641	*Bone morphogenetic protein receptor type-2 precursor*
		R-AGTCCAAAGAGAATCAGAGTTC								
2A1340	2	F-TTGGAGAGGCAGAATTAACTTG	(AAAG)^15^	17	296–321	7	0.713	0.529	0.025∗	*Metallopho phoesterase domain containing 2*
		R-CATGTAAATGTGAGCAAACCAC								
2A2910	2	F-CATCAATTTATCCTCCACCCTC	(AAAC)^8^	24	318–335	5	0.696	0.708	0.974	NA
		R-ATTTGTAGCTTGGGTTTGTCTC								
3A2393	3	F-CCCAGAAACTTAGAAGCTAGTG	(AGAT)^8^	21	244–260	8	0.828	0.667	0.172	*Cysteine conjugate-beta lyase 2*
		R-CATTAGAGTGTCACGGAAGAG								
4A2131	4	F-ATTTCCATTCCAGAATCTCCAC	(ACTC)^10^	23	134–156	9	0.831	0.783	0.802	*Transmembrane protein 246*
		R-TTGTTTAAAGGTGCAAGGTTTG								
5A672	5	F-AAAGGTTTACAACTCCATACCC	(AGAT)^12^	24	230–346	5	0.751	0.75	0.839	NA
		R-GAAGGAGTAAGATGCACAGAAC								
5B59	5	F-ATGCTGGTATTGTGTAGGATTG	(AAAG)^21^	24	186–207	17	0.918	0.833	0.435	NA
		R-TTAGTGTAGAGGAATGAGAGGC								
6A496	6	F-GTAAAGTTGTGCAATGTCAGC	(AGAT)^15^	23	437–457	6	0.82	0.913	0.941	*Neurexophilin 1*
		R-TATAATGTCCTAGCTCTGTAGG								
6A565	6	F-AGTTAATTCAGTGCTTGTTGGG	(AGAT)^18^	24	246–318	9	0.862	0.708	0.028∗	NA
		R-ATCTGATCTCCTCTTCTGTCAG								
8A401	8	F-TCAACACTTTCGAGGTTTAGTC	(AAAC)^8^	24	352–368	4	0.574	0.5	0.725	*Coiled-coil domain containing 130*
		R-CTTTGCTTTGATTGTGACCATG								
8A1226	8	F-TCATTCCATTTCCAACTCAGAC	(AGAT)^12^	24	418–422	2	0.422	0.417	1	NA
		R-CTTTGCTTTGATTGTGACCATG								
8A472	8	F-AAAGGGAGGAGGAAGAAAGAAC	(ACAT)^9^	22	349–403	19	0.947	0.864	0.012∗	NA
		R-CCATTAGCACCATCTCTATTCG								
9A1141	9	F-GATCTGGTCTGAGTTGTCTG	(AACT)^12^	21	227–235	3	0.528	0.238	0.007∗	*Centrosomal protein 70*
		R-CCATTAGCACCATCTCTATTCG								
9B878	9	F-AAGAGACAGTATTGAAAGCATG	(AGAT)^9^	24	407–439	9	0.87	0.75	0.422	*Olfactory receptor 904*
		R-AGCTGAATTTACTCCAAGCATC								
10B1562	10	F-CAGCACTAAACCTAACTACACC	(AGAT)^11^	22	427–428	2	0.304	0.182	0.106	*Transmembrane and tetratricopeptide repeat containing protein 2*
		R-AGCTGAATTTACTCCAAGCATC								
11A2041	11	F-TCTAAATTCTTGATGCACCTGG	(AATG)^8^	9	348–372	7	0.81	0.667	0.1563	NA
		R-AGCTGAATTTACTCCAAGCATC								
12A1292	12	F-TCATCTATTGATTGATCCACCC	(AGAT)^9^	20	272–315	14	0.933	0.9	0.540	*A kinase (PRKA) anchor protein 6*
		R-CAGATATAGACACGGAGGTAGG								
12A1851	12	F-CCACCCTTCCATCTATTCATTC	(ACAT)^10^	21	329–369	10	0.835	0.381	0∗	*Family with sequence similarity 181, member A*
		R-CAGATATAGACACGGAGGTAGG								
14A594	14	F-CCTATGGAAGCTTTGTGAGTTG	(ACAG)^8^	22	445–471	12	0.915	0.909	0.927	*Potassium large conductance calcium-activated channel, subfamily M, alpha member 1*
		R-ATAATTCAACCAAACCGTGTCC								
15B141	15	F-CAAGAACAGGAGAAGAGTCAAG	(AAGG)^10^	21	410–419	3	0.354	0.333	1	*Zinc finger protein 706*
		R-TATATTCAACTGAGTCACTGCC								
15B636	15	F-CACAAGTGTAAGGGTATTGG	(AGAT)^13^	24	310–314	4	0.301	0.25	0.093	*Ubiquitin protein ligase E3 component n-recognin 5*
		R-CTAGGGACAATGAACTGACATG								
17A410	17	F-ACATATCTAGTTTCAAGCCAGC	(AAAC)^9^	19	172–181	7	0.858	0.842	0.410	*Meiosis-specific with OB domains*
		R-CAAGTCTCATTGGGTCTATCTG								
17A501	17	F-AGAGAATACAATATGGCACTGC	(ACAT)^10^	18	348–371	9	0.867	0.889	0.536	*Regulator of microtubule dynamics 2*
		R-CAAGTCTCATTGGGTCTATCTG								
18A352	18	F-CCAAATTTAAAGGGAGGCAATG	(AAGG)^12^	24	277–286	2	0.254	0.042	0.001∗	*Mucosa associated lymphoid tissue lymphoma translocation gene 1*
		R-CAAGTCTCATTGGGTCTATCTG								

Ch indicates the chromosome (of *Mus musculus*) where the locus is located. N, Size, N_A_, H_E_ and H_O_ indicate sample size of *A. semotus*, size range of amplified fragments, number of alleles, expected and observed heterozygosity, respectively. P_HWE_ indicates p value of testing for deviation from Hardy-Weinberg equilibrium (HWE), where

^*^indicates deviation from HWE (p < 0.05). Protein coding gene indicates the closest blasted gene within a distance of 100 kbp from the microsatellite, where NA indicates that no gene is identified in the 200-kbp genomic region because there is no blasting result fitting the criteria (Method; see Supplementary Fig. S1 for relative locations of the microsatellites and corresponding protein coding genes).
