# Corrigendum: Developing informative microsatellite markers for non-model species using reference mapping against a model species’ genome

**DOI:** 10.1038/srep45419

**Published:** 2017-06-19

**Authors:** Chih-Ming Hung, Ai-Yun Yu, Yu-Ting Lai, Pei-Jen L. Shaner

Scientific Reports 6: Article number: 23087; 10.1038/srep23087 published online: 03 15 2016; updated: 04 19 2017.

The original version of this Article contained an error in the title of the paper, where the word 'markers' was incorrectly given as 'makers'.

In addition, this Article contained typographical errors in Table 1. In Table 1, the primer sequences for loci 8A1226, 9A1141, 10B1562, 11A2041, 12A1851, 17A501 and 18A352 were incorrect.

These errors have now been corrected in the HTML and PDF versions of the Article.

