# Supplementary material for: Developing informative microsatellite makers for non-model species using reference mapping against a model species’ genome
Source: Sci Rep. 2016 Mar 15;6:23087. doi: 10.1038/srep23087 (PMC4791680; doi:10.1038/srep23087)
Supplement: Supplementary Information [file srep23087-s2.pdf]

**Title: Developing informative microsatellite makers for non-model species using reference mapping against a model species' genome**

Chih-Ming Hung[1], Ai-Yun Yu[2], Yu-Ting Lai[2] and Pei-Jen L. Shaner[2*]

Supplementary materials:

Supplementary Figure S1

Supplementary Table S2

**Supplementary Figure S1.** Map Viewer results of *Mus musculus* (the upper part) and BLASTX results, which comparing *Apodemus semotus* DNA sequences against *M. musculus* protein sequences (the lower part). In the Map Viewer results, digits along the X-axis indicate the locations in the corresponding *M. musculus* chromosomes, horizontal green lines indicate introns and short, vertical green lines indicate exons. Gene names are shown next to the green lines. Purple lines indicate predicted or uncharacterized genes (labeled as "Gm", "LOC" or "Rik") or microRNA (labeled as "Mir"). Long, vertical red lines indicate the locations of the microstatellite loci, and the names of the loci are shown above the red lines. Rectangles indicate 10 kbp segments along *A. semotus* chromosomes anchored on the microstatellie loci. Empty rectangles indicate no BLASTX coding regions found in that 10 kbp segments, whereas blue or black ones (used alternatively for distinguishing different blasted genes) indicate positive BLASTX coding regions located in that 10 kbp segments with gene names shown next to them.

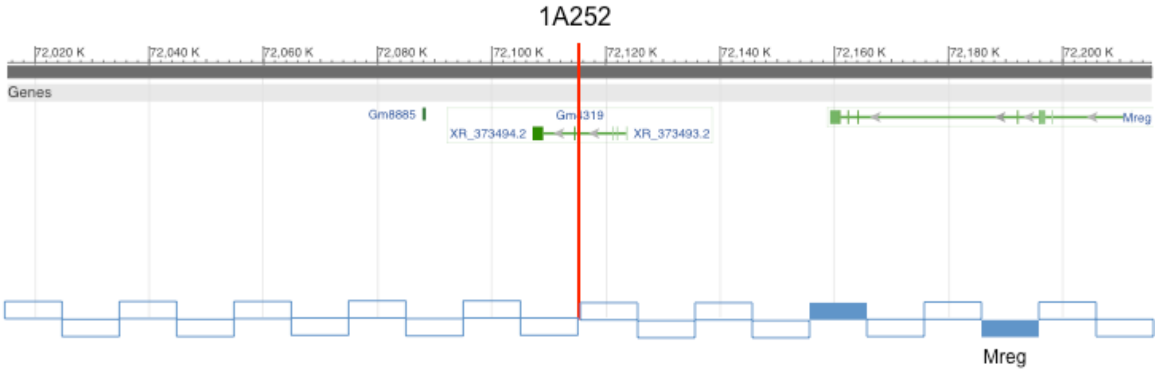

1A720

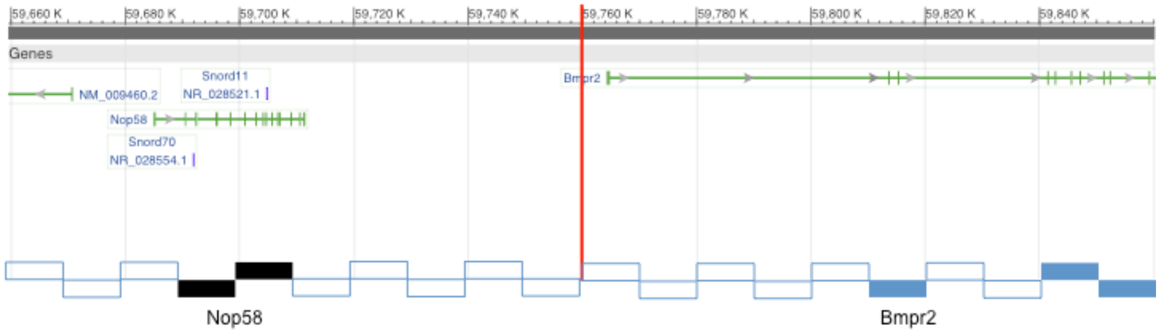


1A1076

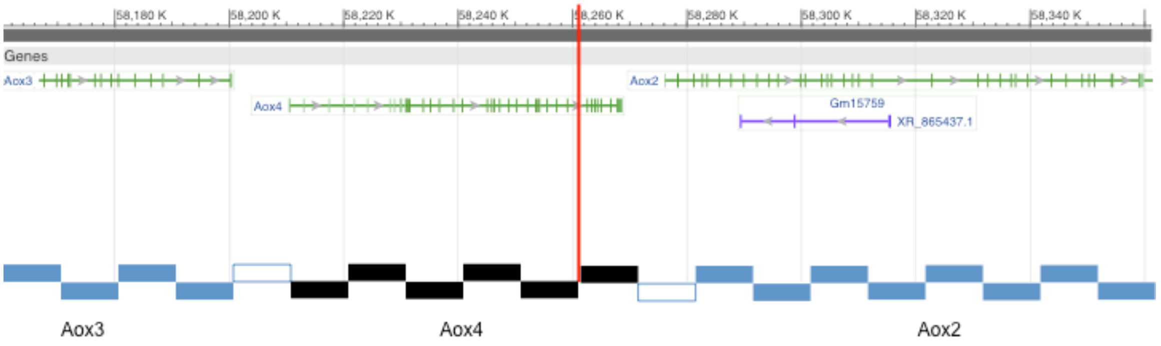


2A1340

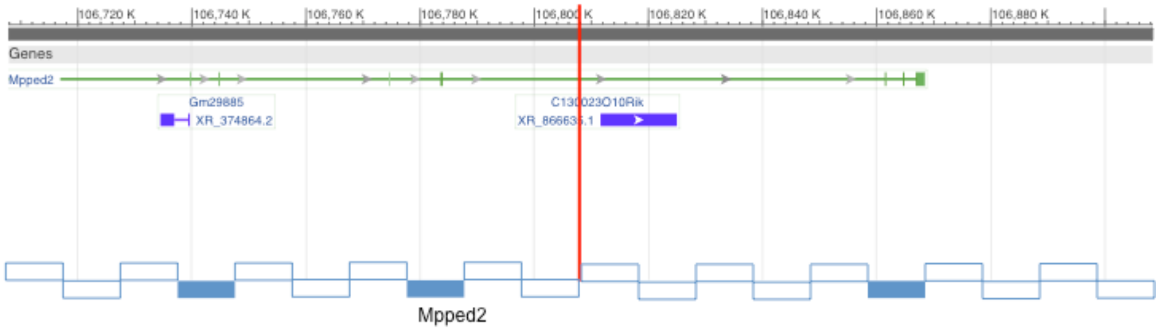


2A1612

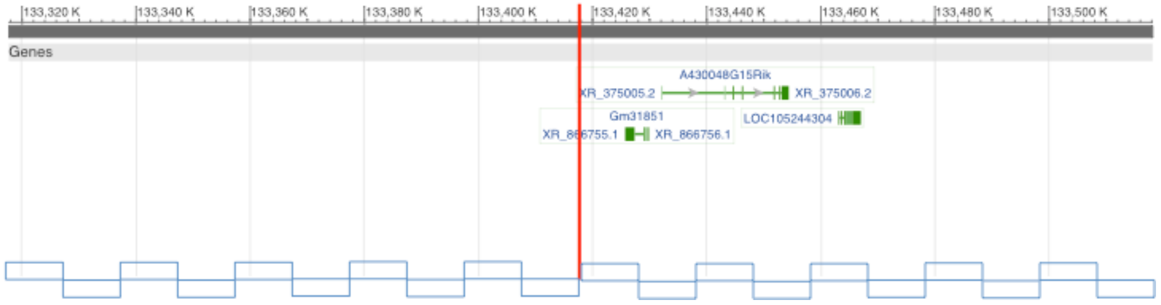

2A2910

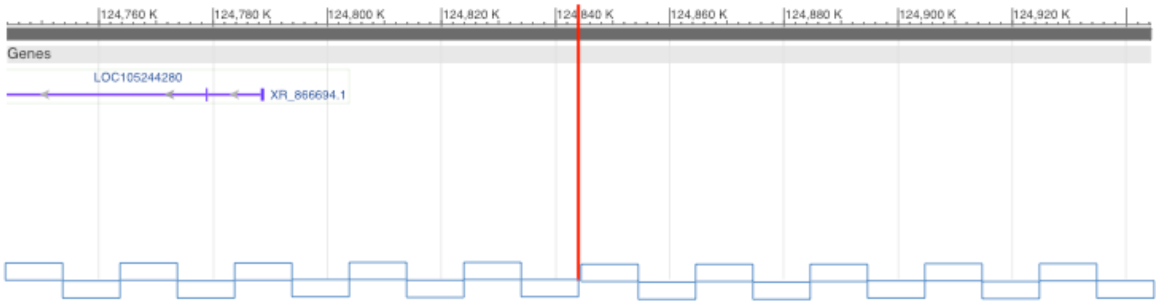


2B430

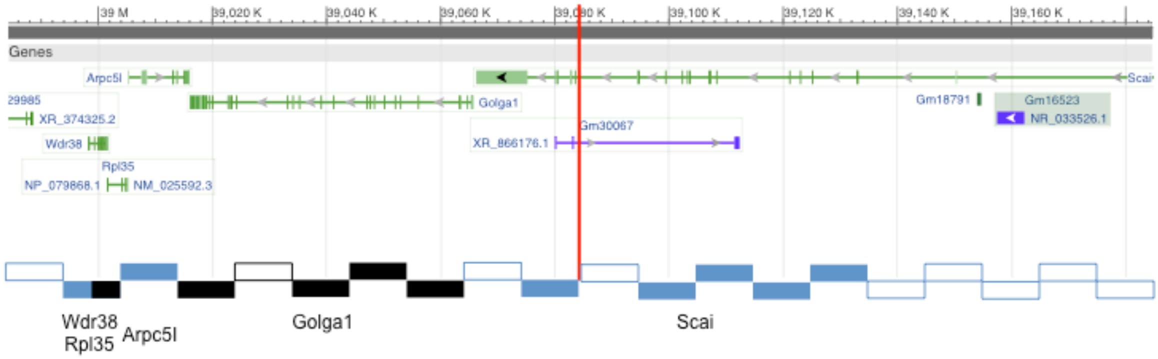


2B756

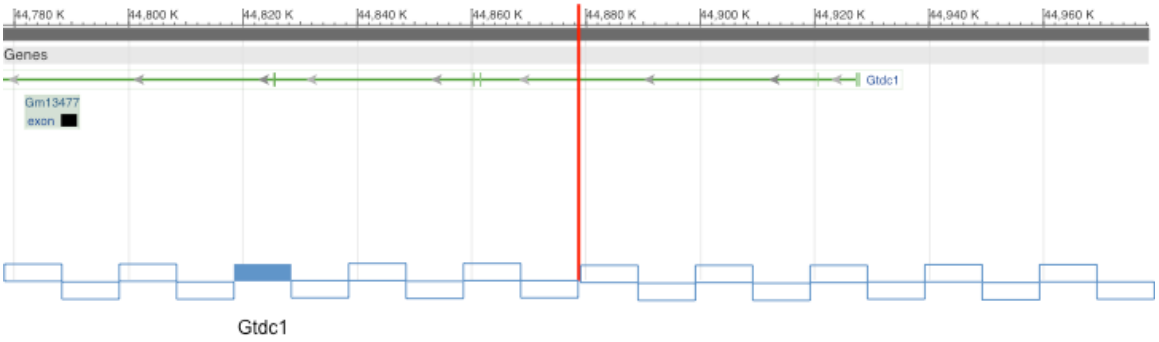


3A1720

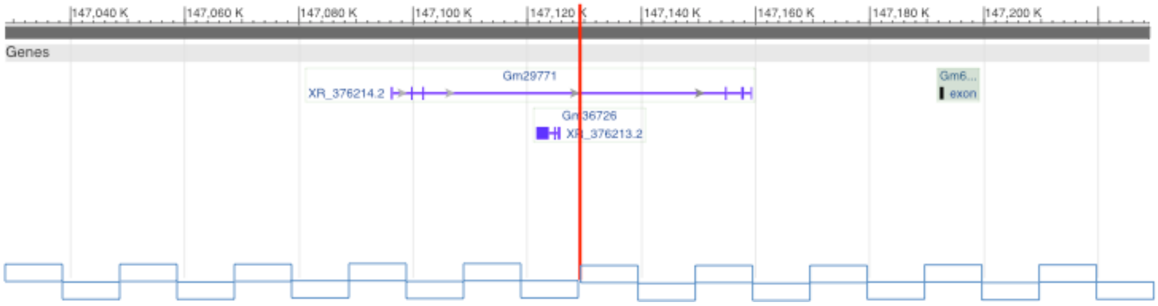

3A2393

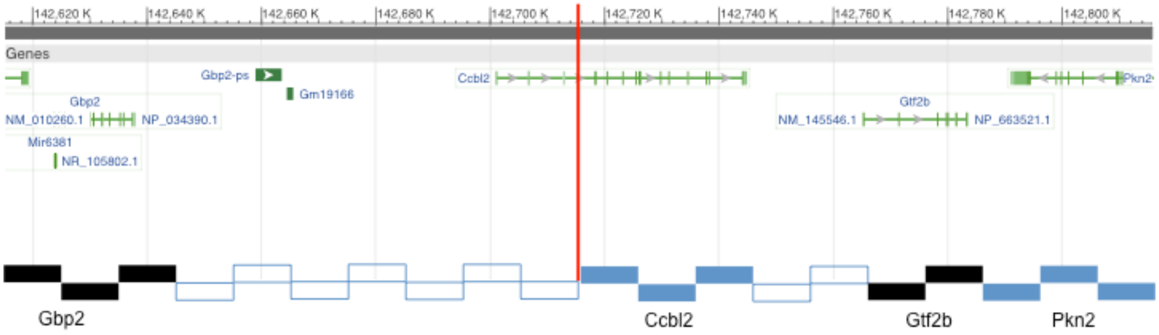


4A2042

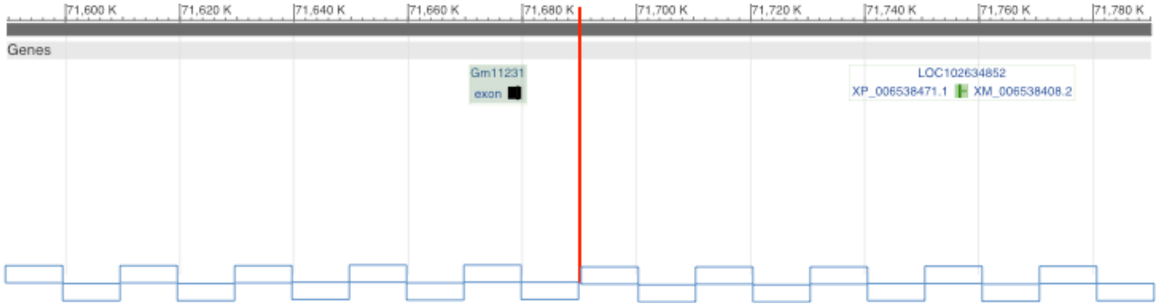


4A2131

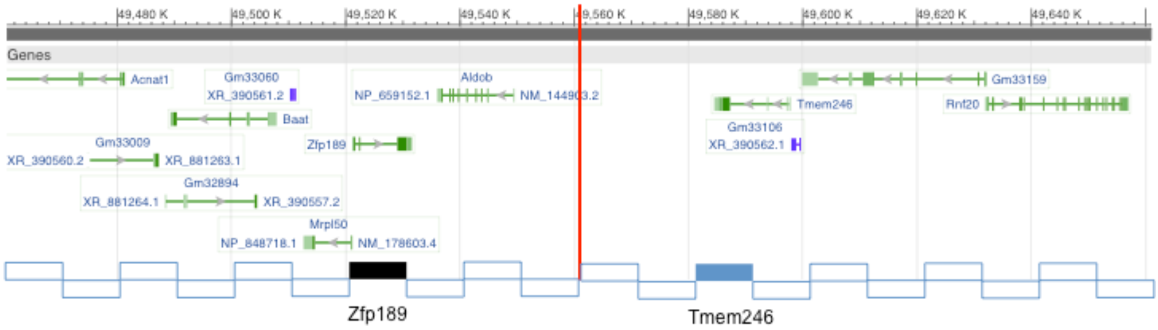


4A2138

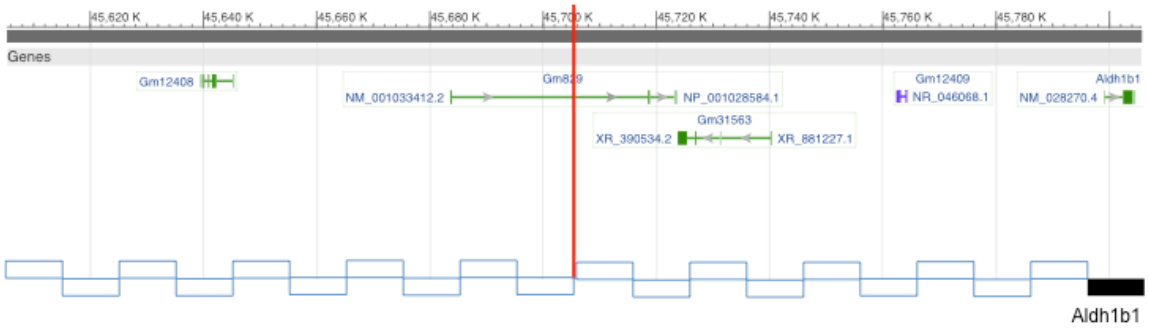

4B263

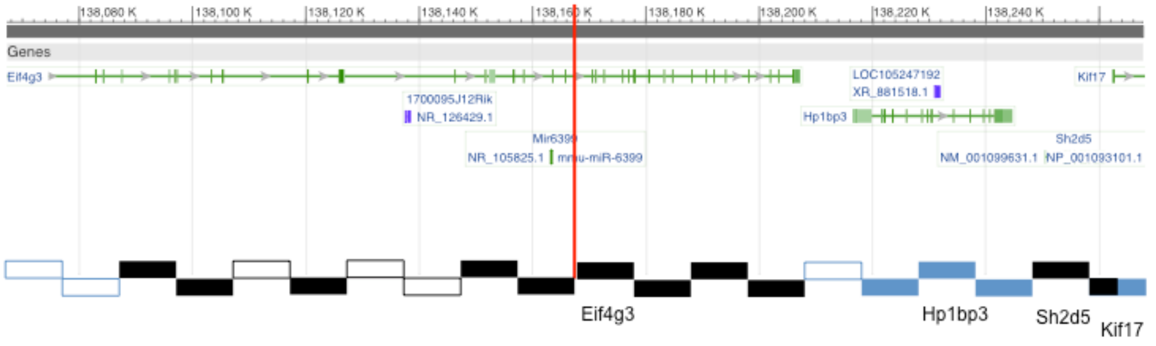


5A296

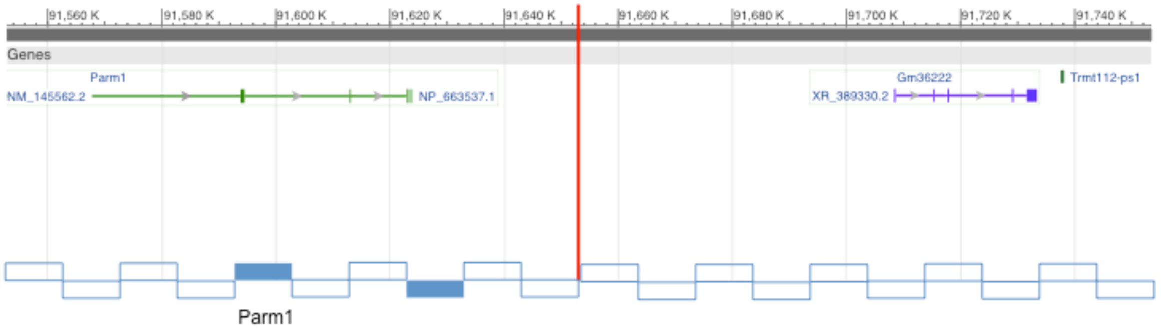


5A672

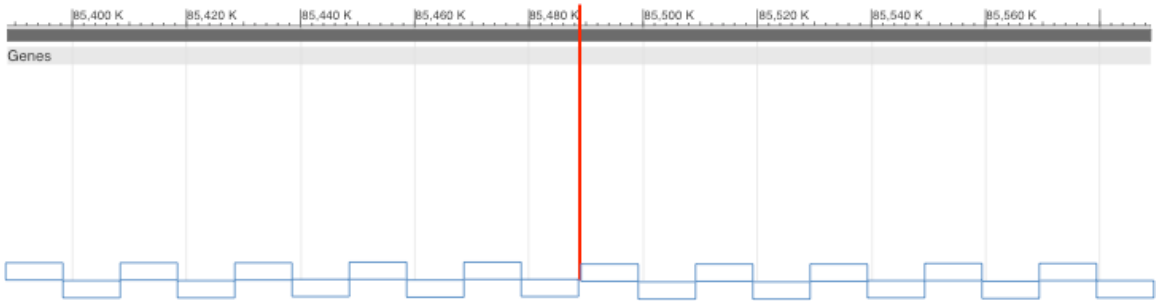


5B59

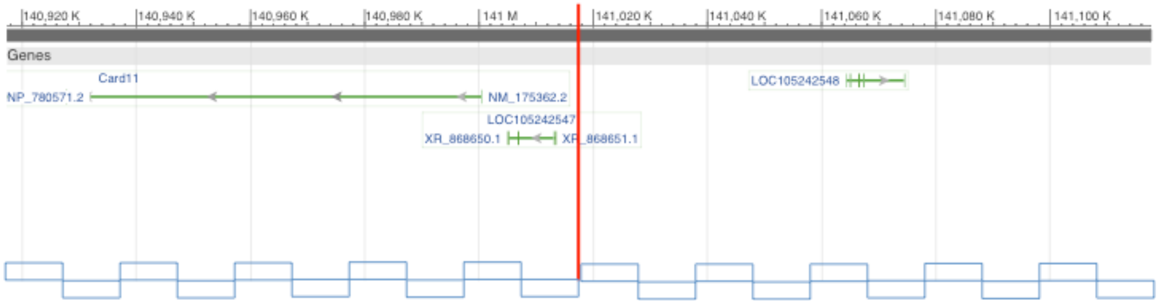

6A496

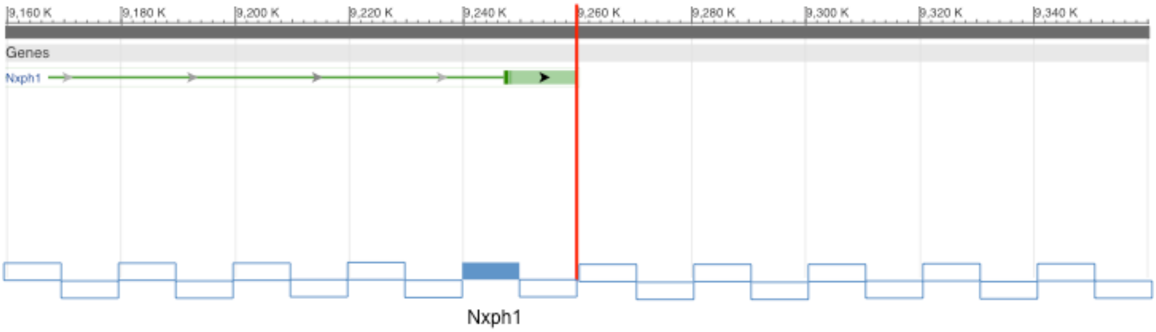


6A565

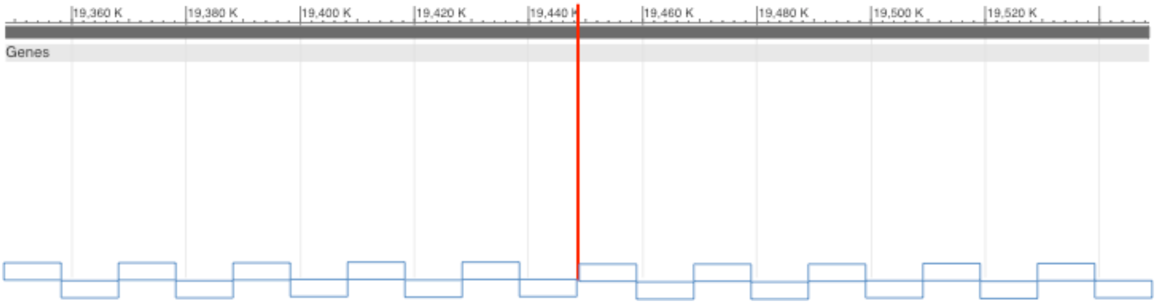


8A401

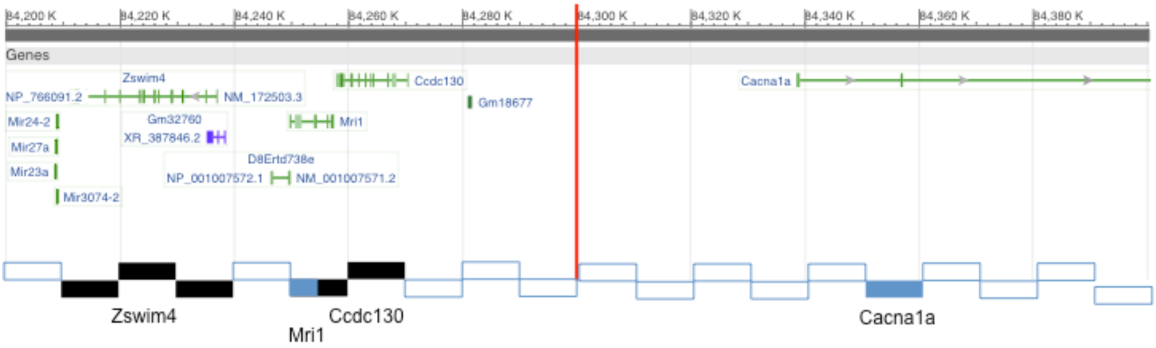


8A472

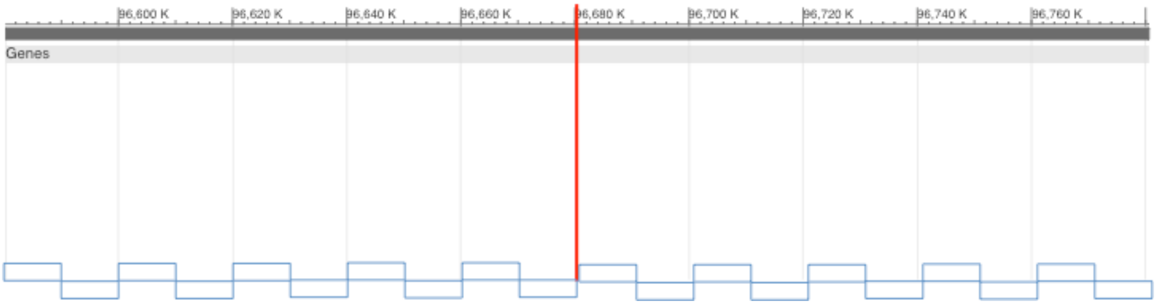

8A1226

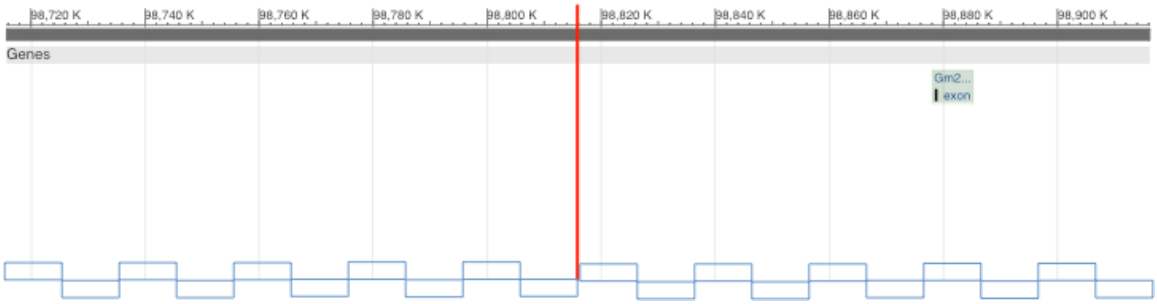


9A300

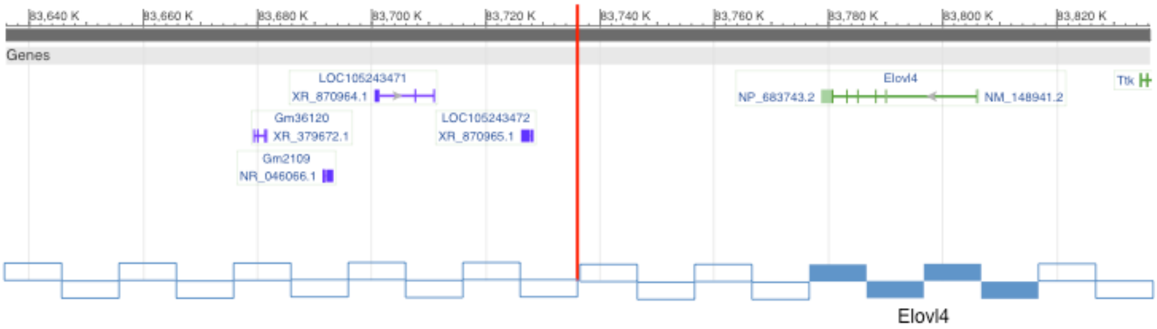


9A338

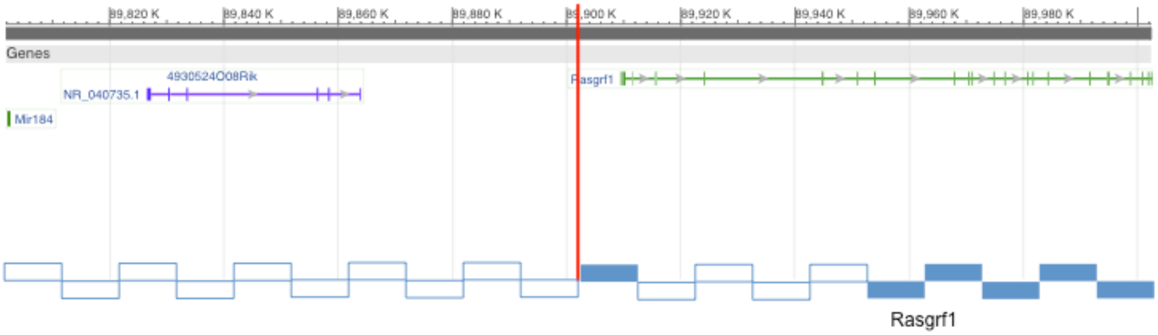


9A1141

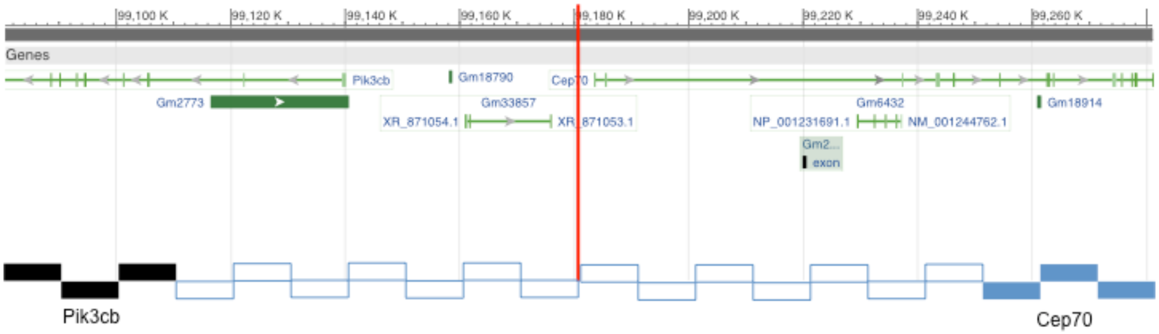

9B815

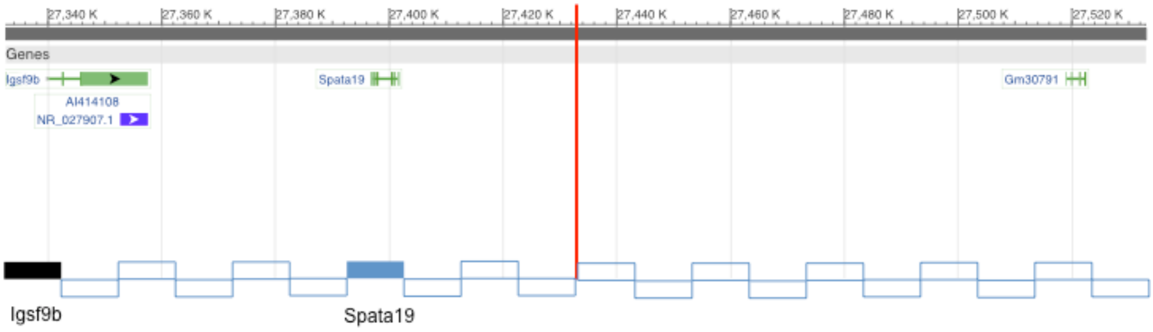


9B878

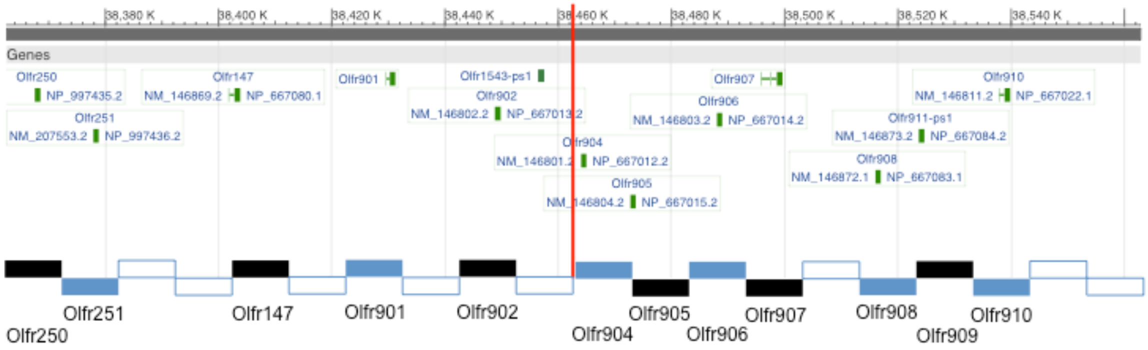


10A44

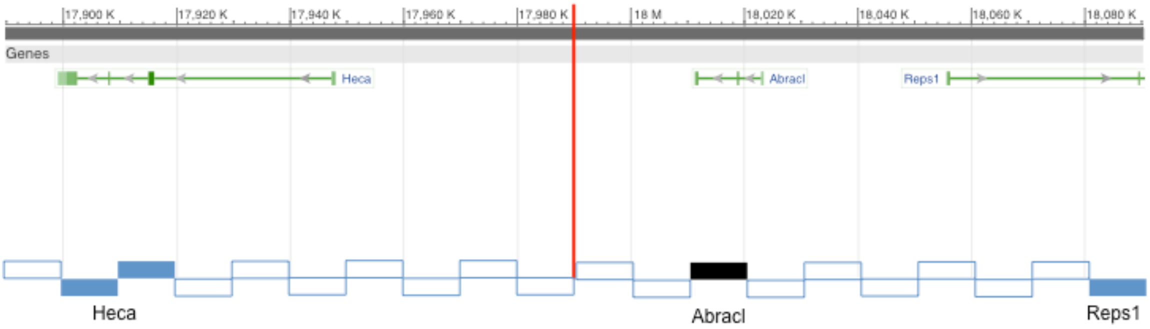


10A378

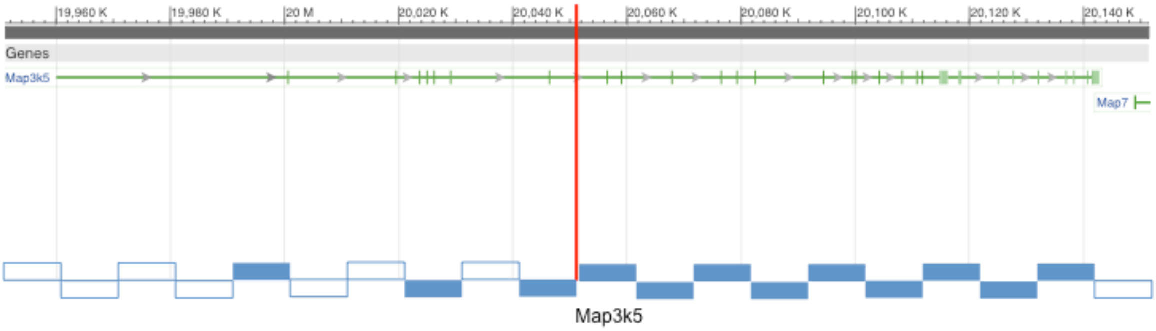

10A496

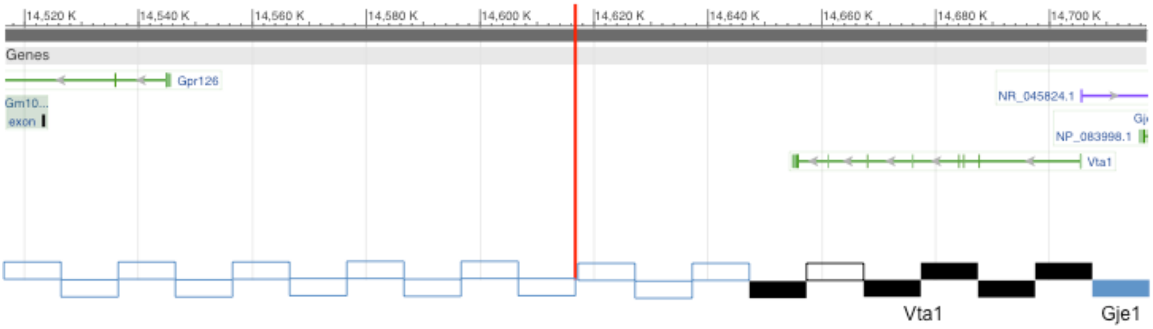


10B1320

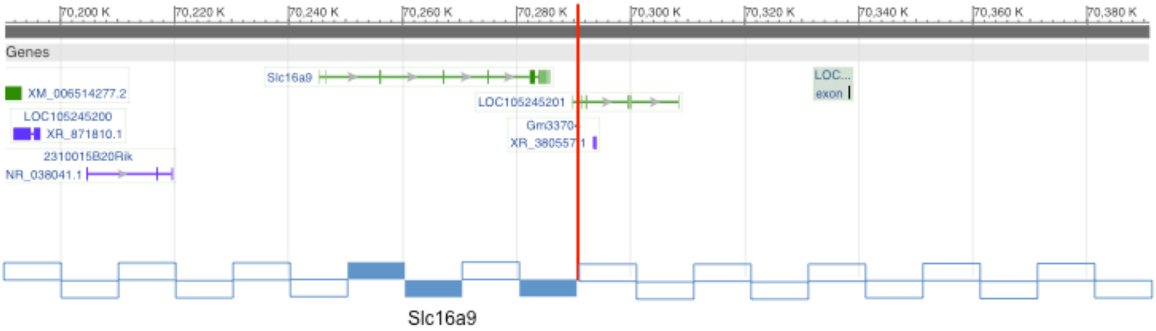


10B1562

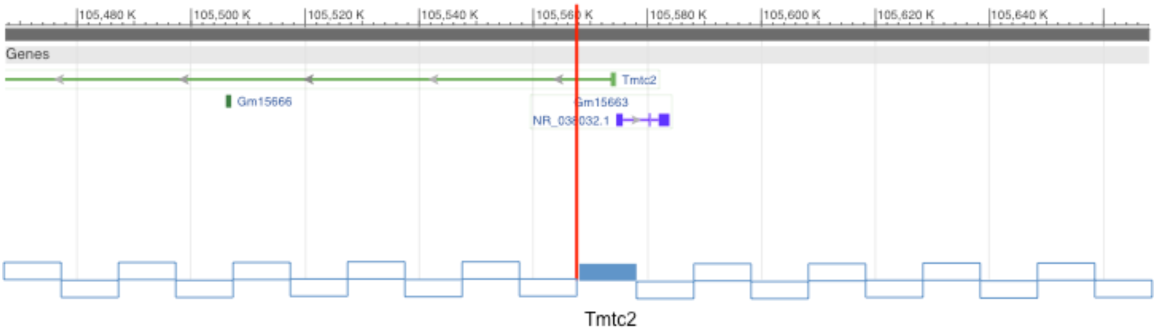


11A172

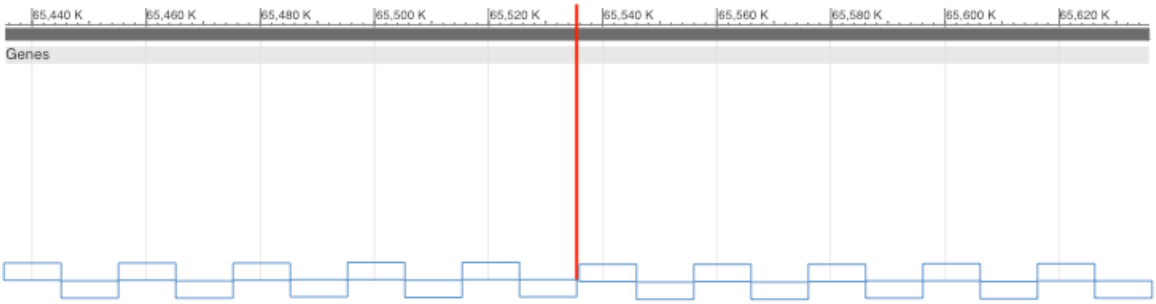

11A2041

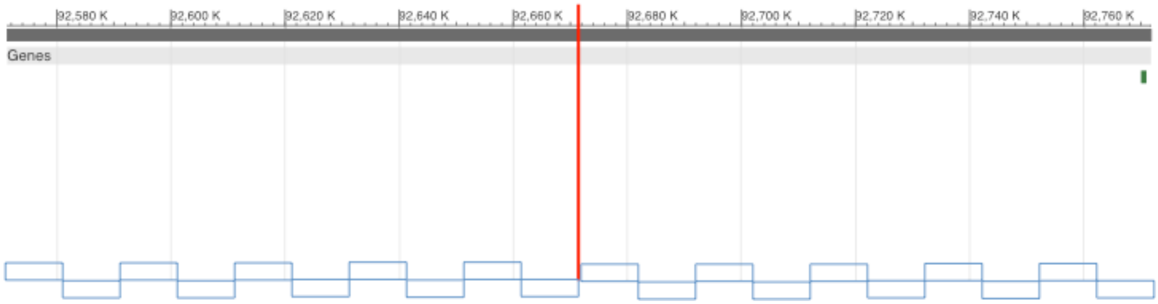


12A204

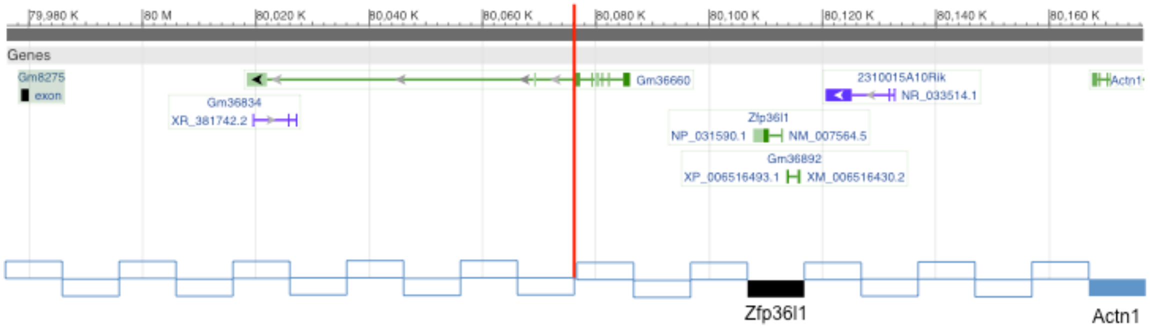


12A1292

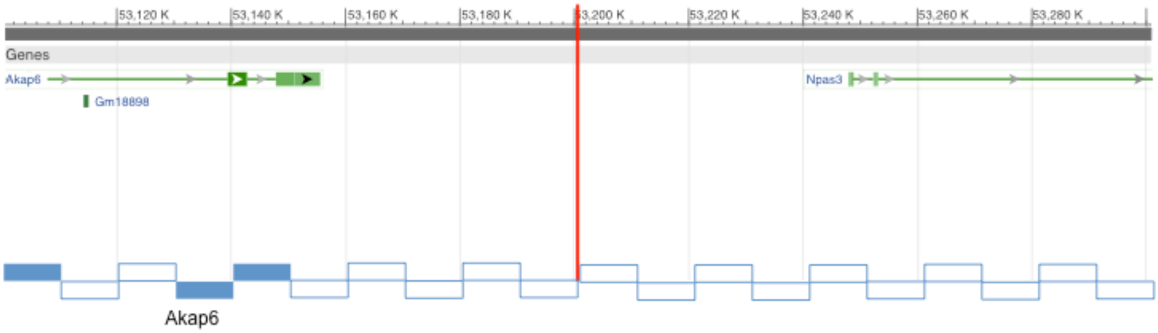


12A1851

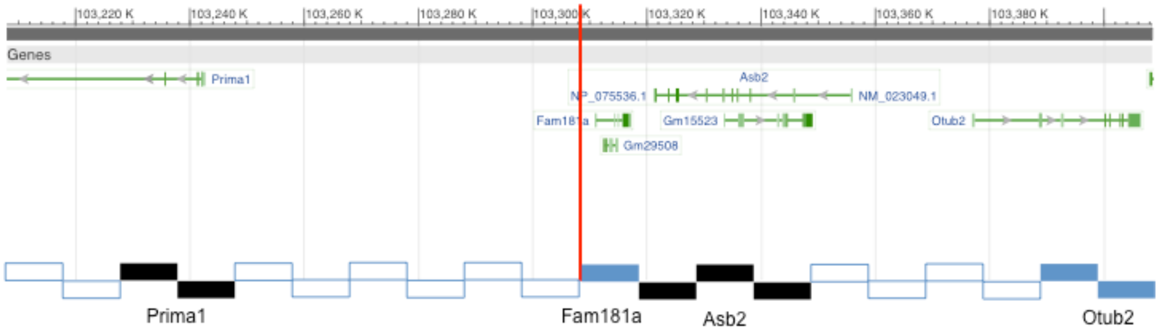

12B30

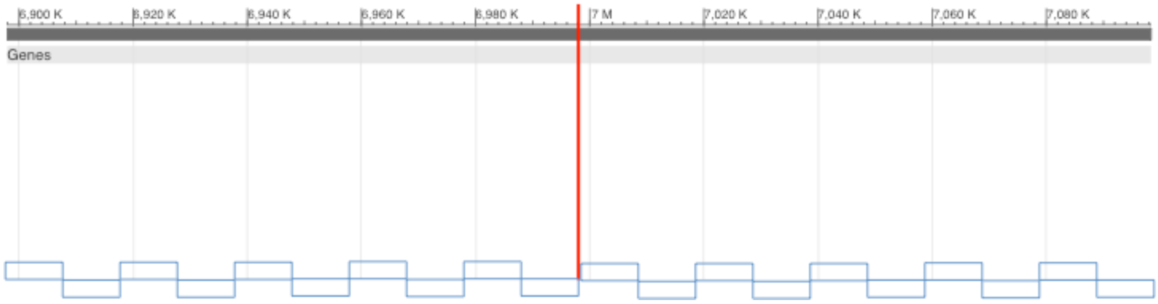


12B908

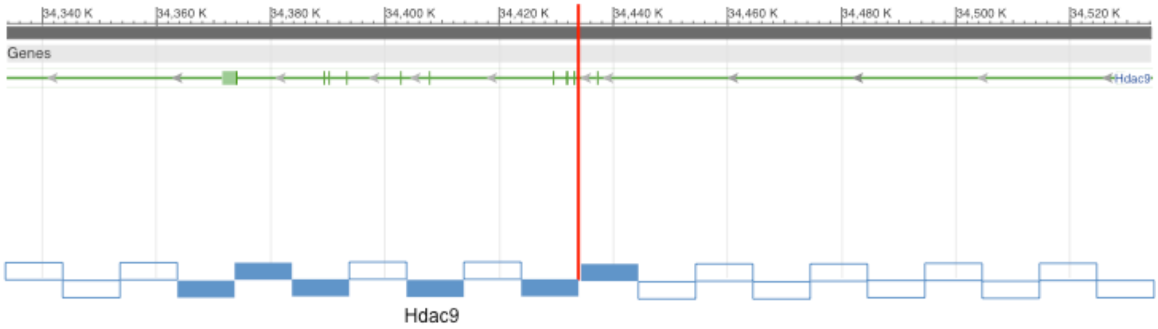


13A191

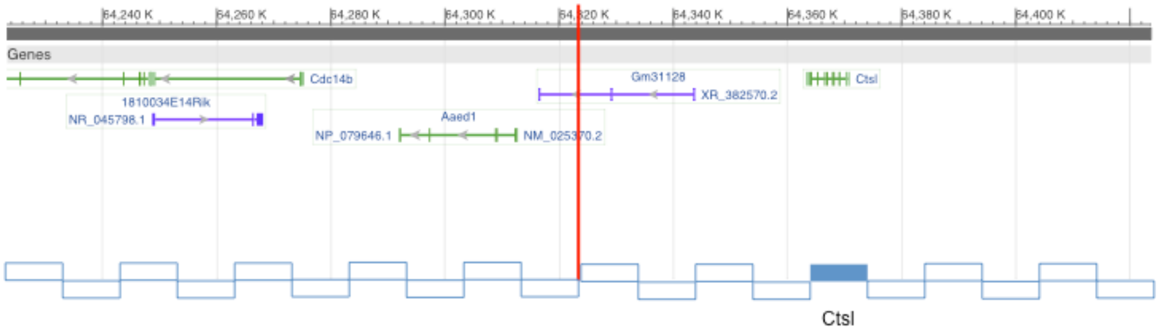


13B150

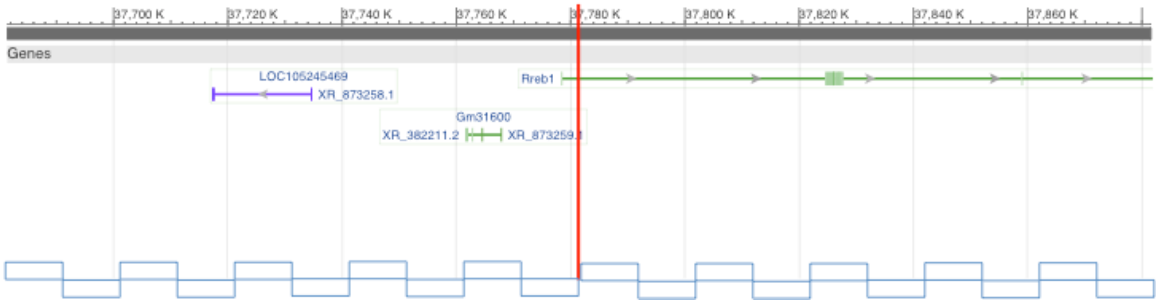

14A594

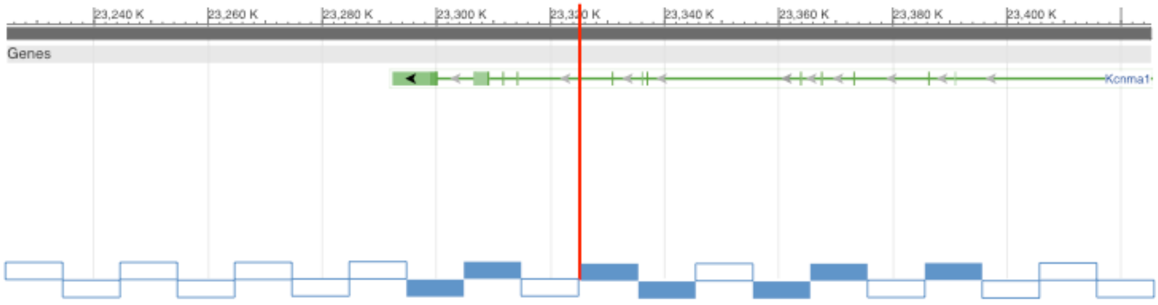


Kcnma1

14A681

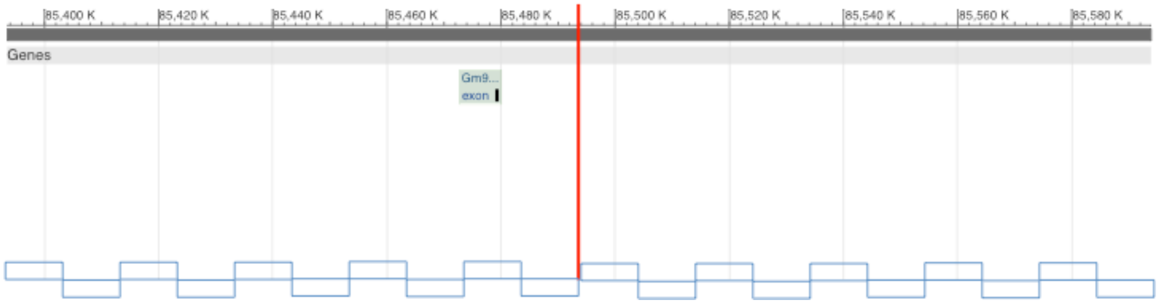


15A446

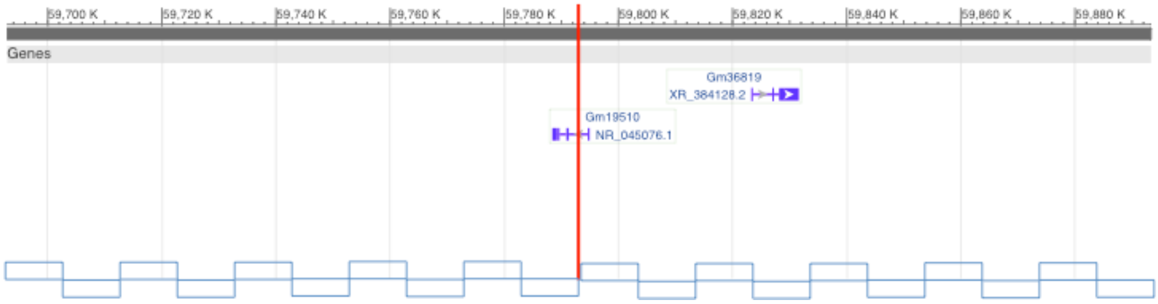


15A493

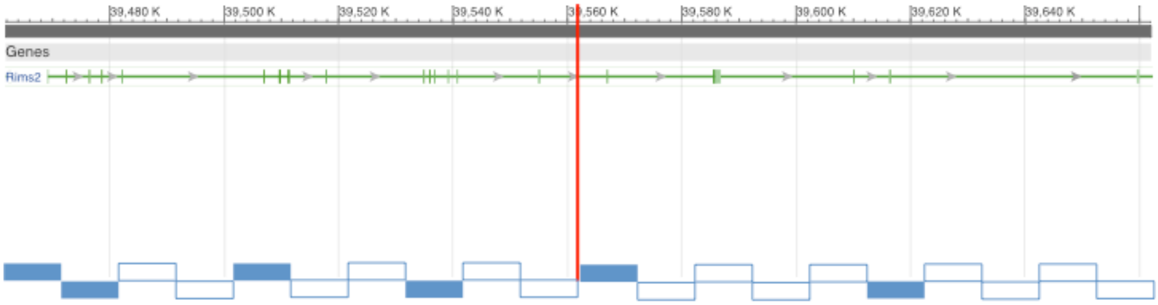


Rims2

15A1106

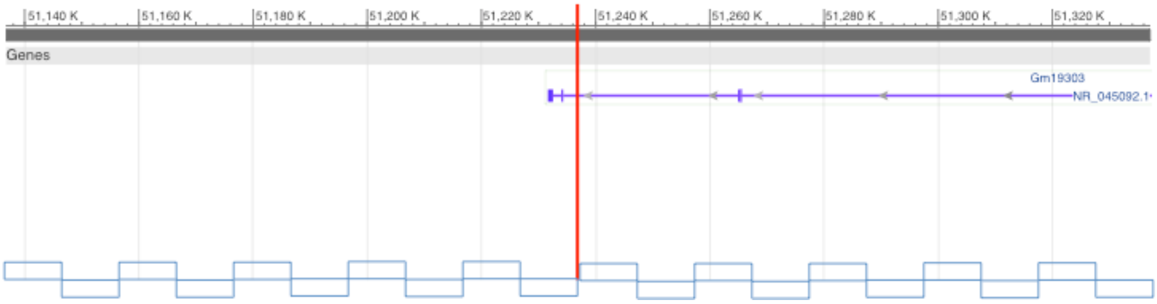


15B141

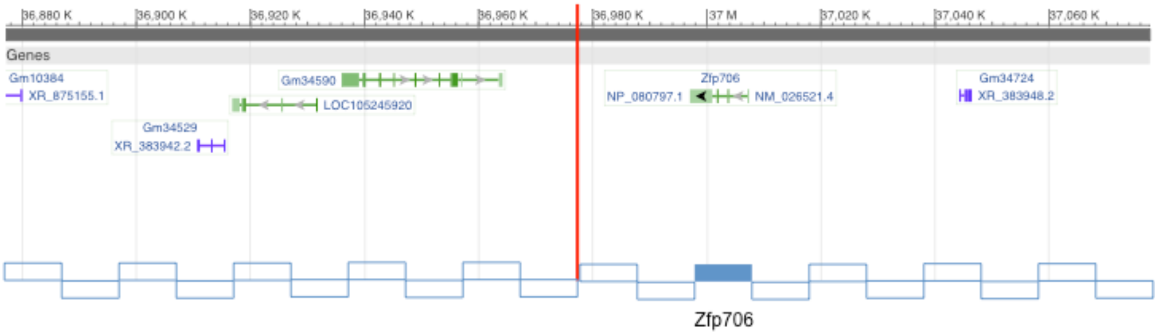


15B636

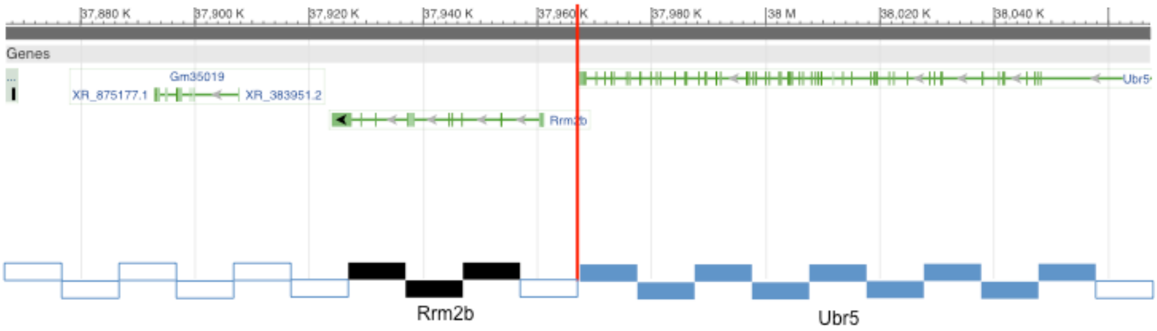


16A373

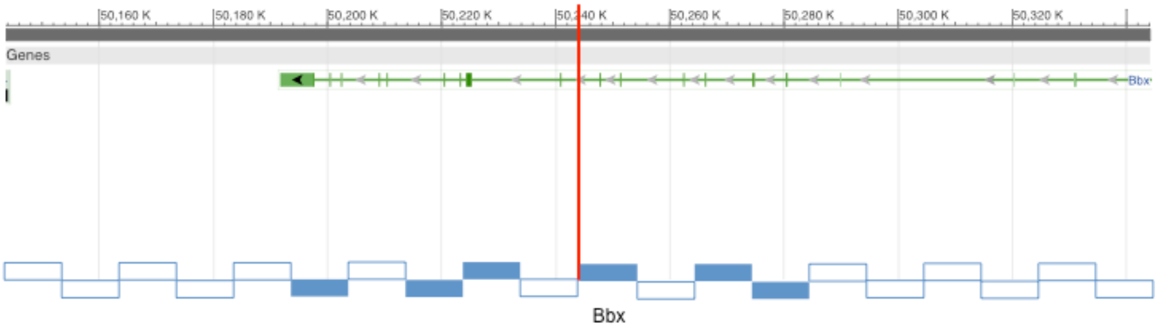

16B785

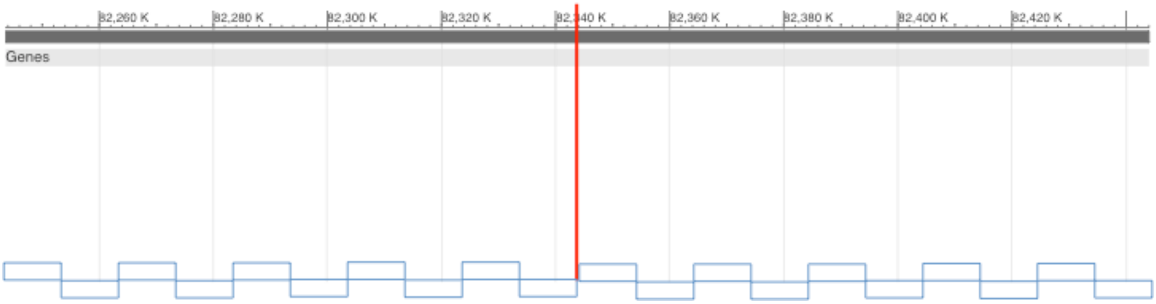


17A352

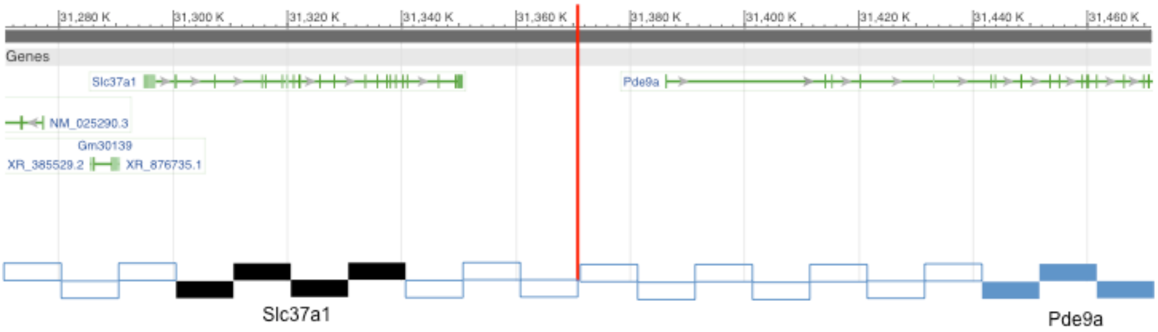


17A410

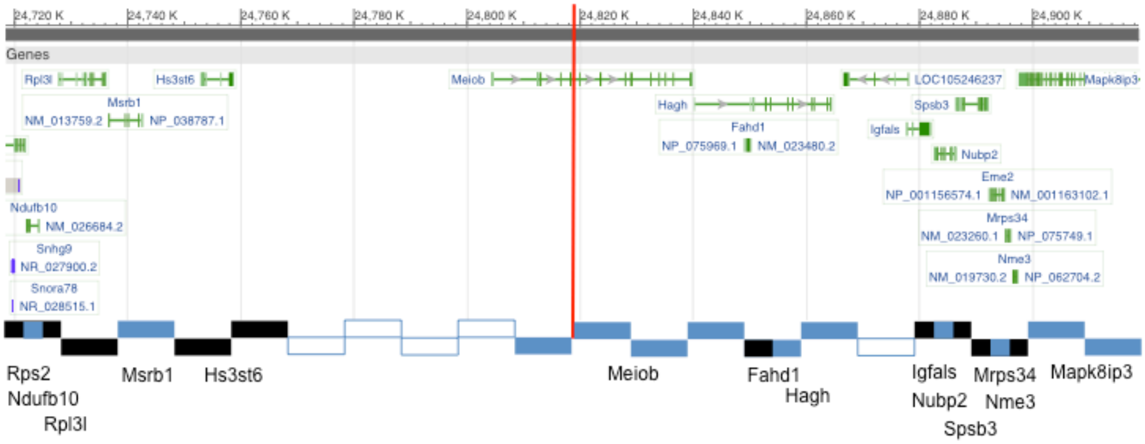


17A501

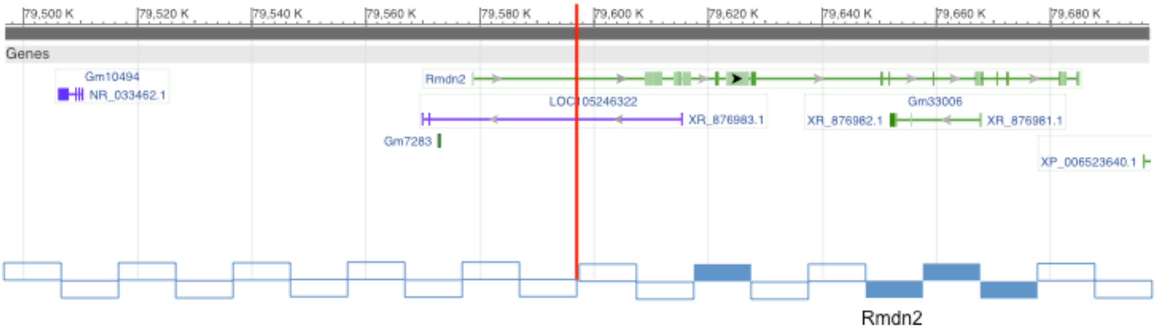

18A352

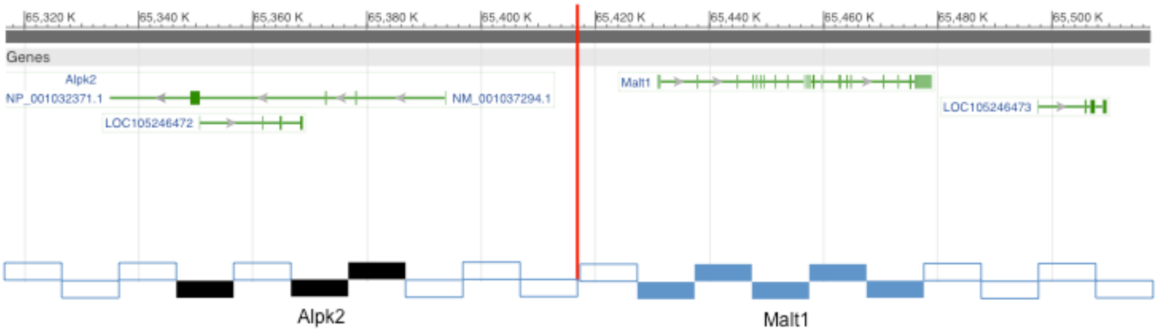


18A506

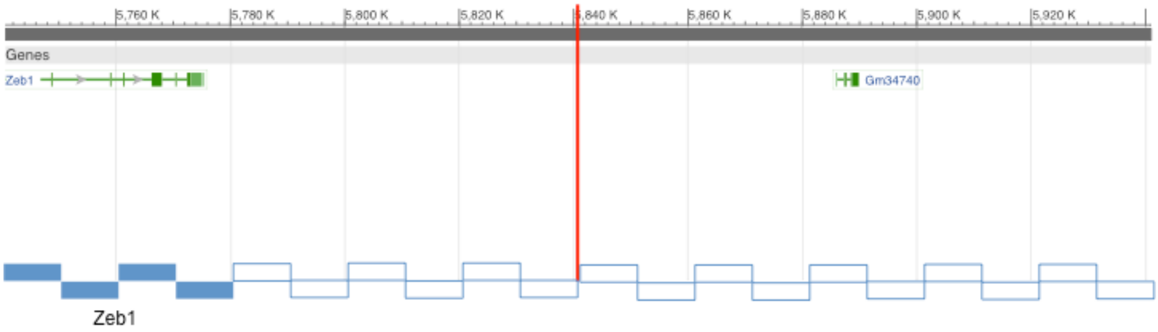


18A1452

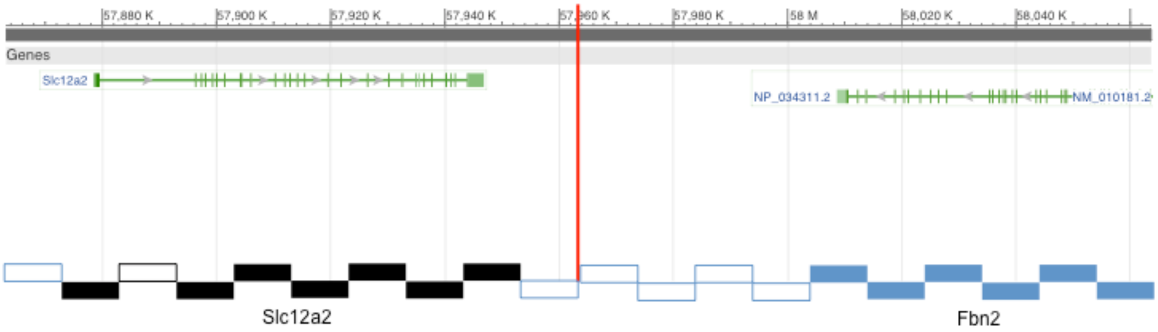


19A40

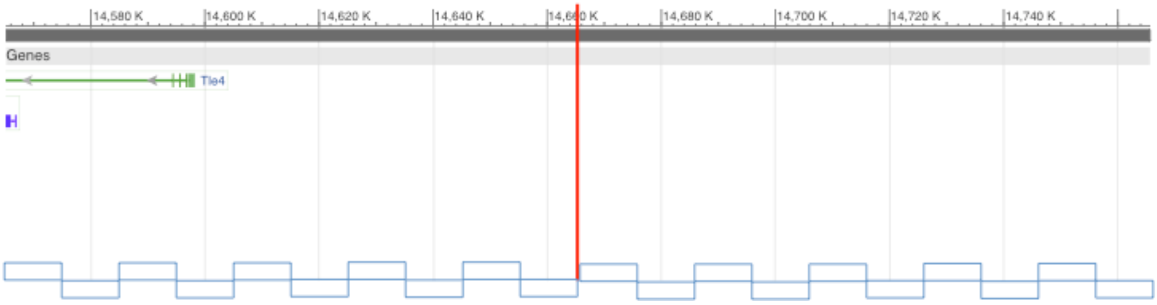

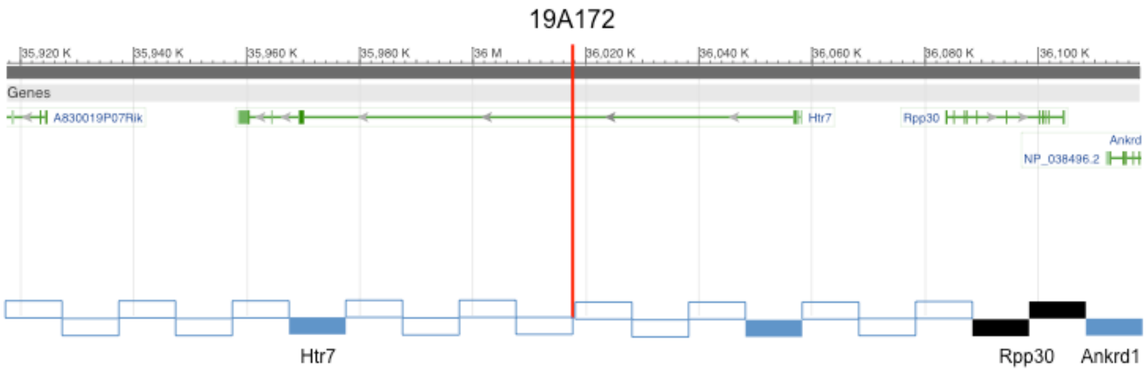


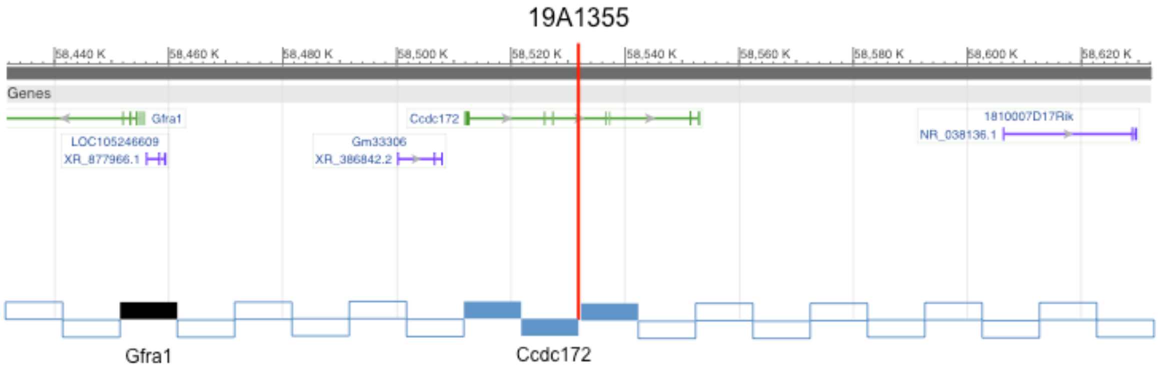


**Supplementary Table 2.** Laboratory cost, time and yield compared between the traditional approaches of microsatellite isolation based on cloning and Sanger sequence[36,37] and our approach based reference genome mapping.

| | Traditional approaches (Cloning + Sanger sequencing) | Our approach (Shotgun sequencing + reference genome mapping) |
|---|---|---|
| Time for bench work | 1 - 4 weeks | 1 - 2 days |
| Cost (USD) | 1,100 – 4,400 | 3,000 |
| Yield (loci) | 100 | 1,456 |
| Cost / locus | 11 - 44 | 2.1 |
